# SMAD4 contributes to chondrocyte and osteocyte development

**DOI:** 10.1111/jcmm.17080

**Published:** 2021-11-28

**Authors:** Katayoon Pakravan, Ehsan Razmara, Bashdar Mahmud Hussen, Fatemeh Sattarikia, Majid Sadeghizadeh, Sadegh Babashah

**Affiliations:** ^1^ Department of Molecular Genetics Faculty of Biological Sciences Tarbiat Modares University Tehran Iran; ^2^ Department of Medical Genetics Faculty of Medical Sciences Tarbiat Modares University Tehran Iran; ^3^ Department of Pharmacognosy College of Pharmacy Hawler Medical University Kurdistan Region Iraq

**Keywords:** chondrogenesis, epigenetic modulations, osteogenesis, signalling pathways, SMAD4

## Abstract

Different cellular and molecular mechanisms contribute to chondrocyte and osteocyte development. Although vital roles of the mothers against decapentaplegic homolog 4 (also called ‘SMAD4’) have been discussed in different cancers and stem cell‐related studies, there are a few reviews summarizing the roles of this protein in the skeletal development and bone homeostasis. In order to fill this gap, we discuss the critical roles of SMAD4 in the skeletal development. To this end, we review the different signalling pathways and also how SMAD4 defines stem cell features. We also elaborate how the epigenetic factors—ie DNA methylation, histone modifications and noncoding RNAs—make a contribution to the chondrocyte and osteocyte development. To better grasp the important roles of SMAD4 in the cartilage and bone development, we also review the genotype‐phenotype correlation in animal models. This review helps us to understand the importance of the SMAD4 in the chondrocyte and bone development and the potential applications for therapeutic goals.

## 1 INTRODUCTION

**FIGURE 1:**
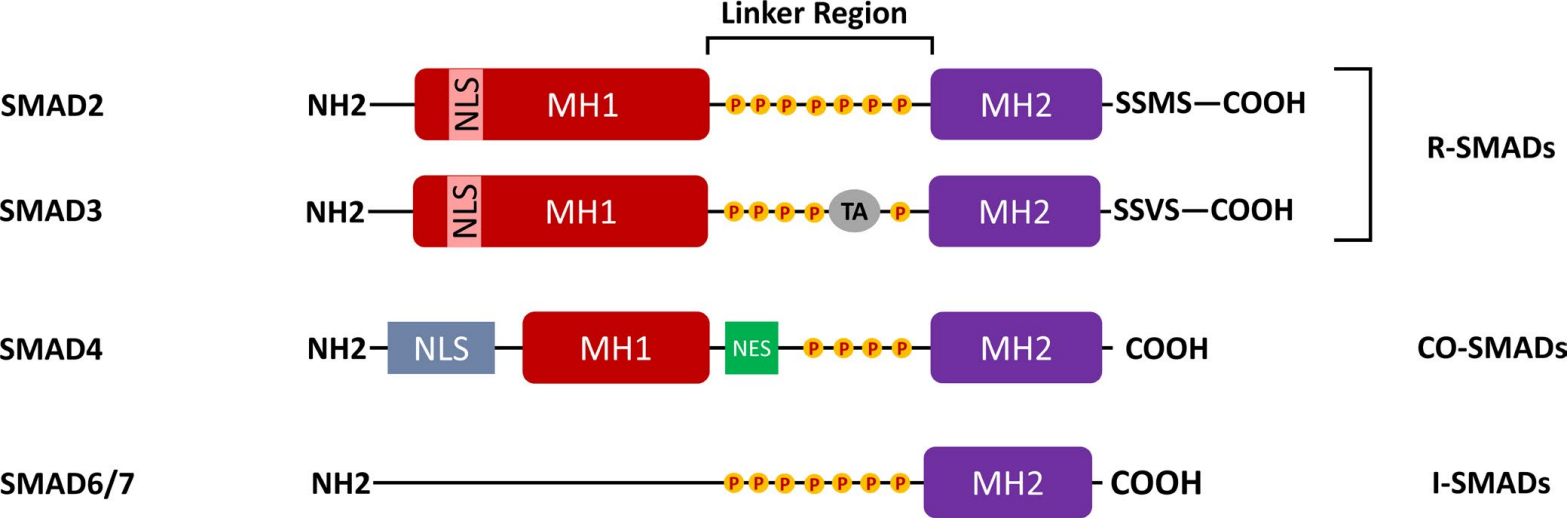
The structure of some important members of the SMAD family. The comparison between the three main classes of SMADs including R‐SMADs, co‐SMAD and I‐SMADs is depicted. The MH1 domain is shown by red and is absent in the I‐SMADs including SMAD6/7. This section is linked to the MH2 region (Purple) by a linker region that can be subjected to phosphorylation. This state can change the functions of putative SMADs. SMAD4 embraces nucleus export signal (NES) in its linker region. Additionally, SMAD2, SMAD3 and SMAD4 contain a nucleus localization signal (NLS) in their MH1 domain. C‐terminal SS‐X‐S motifs are present in R‐SMADs. The figure is redrawn from Ref. [10, 140]

The formation of skeletal elements often follows a basic path that involves different chondrogenic and osteogenic programmes.^1^ Among these, a significant number of signalling pathways, eg SMAD family, play roles. The term ‘SMAD’ was coined from a combination of a gene name from *Caenorhabditis elegans* SMA ("small" worm phenotype) and MAD family ("mothers against decapentaplegic") of genes in *Drosophila melanogaster*.^2^ By considering their functions, eight members of SMAD proteins are categorized into three main classes including (i) receptor‐regulated or regulatory SMADs (also known as R‐SMADs), ie SMADs 1, 2, 3, 5 and 8, (ii) common SMAD or co‐SMAD that only involves SMAD4 and (iii) inhibitory SMADs (I‐SMADs) that contain SMAD6 and SMAD7 (reviewed in Ref. [3]) (Figure 1).

SMADs play the fundamental roles in cell signalling during the skeletal development (from chondrocyte precursors to mature osteocytes).^3^ However, the underlying molecular mechanisms whereby the SMADs (especially SMAD4) exert their functions in these procedures are yet unclear. SMAD4 was first investigated in the context of transforming growth factor‐β (TGF‐β) family signal transduction.^4^ In general, by the time TGF‐β upstream signals stimulate cell signalling, SMAD4 interacts with R‐SMADs (ie SMAD2/3) and subsequently forms an oligomer complex that modulates the expression of target genes.^4^ By interacting with SMAD1, 2, 3 and 5, SMAD4 is actively involved in the intracellular signalling pathways of all three types of TGF ligands.^5^ Aberrant expression of *SMAD4* affects the normal TGF‐β signalling and leads to the uncontrolled cell growth and tumour induction in different tissues.^5^, ^6^, ^7^, ^8^, ^9^ This, therefore, shows the paramount importance of SMAD4 in cell growth and development.

SMAD family has similar structures in general. They are composed of two important conserved domains including N‐terminal Mad homology domain‐1 (MH1 domain) and the C‐terminal Mad homology domain 2 (MH2 domain).^10^ While DNA‐binding activity is often attributed to the MH1 domain, the MH2 domain fulfills some transcriptional activities.^11^ These two domains are connected each other by a linker region (a regulatory region in which the different phosphorylation signatures are located) that in turn changes the SMAD4 functions^12^ (Figure 1). R‐SMADs have a short conserved pattern of two serines separated by another amino acid (Ser‐X‐Ser). After phosphorylation,^13^ this sequence activates R‐SMADs, but this section is absent in SMAD4 (Figure 1).

During the development, SMADs contribute to primitive streak formation,^14^ neural crest migration,^15^ gastrulation,^16^ left‐right asymmetry,^17^ self‐renewal of hematopoietic stem cells^18^, ^19^ and morphogenesis of different tissues.^20^, ^21^ These processes cover some aspects of the chondrocyte and osteocyte development. Axiomatically, the SMAD family—especially SMAD4—may play the important roles in the skeletal development and tissue homeostasis. In order to show how SMAD4 plays a role during the cartilage‐to‐bone development, herein, we discuss the molecular mechanisms mediated by this protein. Furthermore, we put forth some information about the SMAD4 epigenetic regulations (eg noncoding RNAs, DNA methylation and histone modifications) during the skeletal development. We also discuss how the genotype‐phenotype correlations of SMAD4 frame our understanding about the complexities of concepts of the skeletal development and also discuss the possible therapeutic applications using this protein.

## 2 SMAD4 MODULATES STEM CELL FEATURES

Migratory neural crest cells follow different pathways to differentiate into neurons, chondrocytes, bones and mesodermal cells.^22^ Therefore, every disrupted signalling pathway may change these cell fates. As the most important precursor for cartilage and bone cells, the mesenchymal stem cells (MSCs) are developmentally traced back to the ‘paraxial mesoderm’ structure^23^, ^24^ that makes cartilages, bone structures and tendons.

Park et al^25^ showed that SMAD4 regulates ‘lineage commitment’ of MSCs by modulating the retention of Taz in the nucleus during the MSC differentiation. A reciprocal role of SMAD4 has been suggested as a positive and negative factor in osteogenesis and adipogenesis of MSCs respectively. An interaction between the Taz and runt‐related transcription factor 2 (RUNX2) subsequently enhances the transcriptional activities of RUNX2^26^ that in turn increases osteogenesis. Furthermore, SMAD3/4 predominantly induces the chondrogenesis of human bone marrow‐derived MSCs.^27^ This can underscore the paramount importance of SMAD4 in inducing the commitment of MSCs, so this explains why the decreased expression of SMAD4 slightly inhibits chondrogenesis and osteogenesis in animal models. Avery et al. demonstrated that the quick differentiation of human embryonic stem cells in response to the inhibition of TGF‐β/Activin/Nodal receptor depended on the presence of SMAD4.^19^ SMAD4 functions in the morphoregulatory networks in turn determine the pattern of different limb bud mesenchymal lineages and/or regulate the tissue differentiation.^28^ SMAD4 in fact functions during the specification and aggregation of the SRY‐box transcription factor 9 (SOX9)‐positive prechondrogenic progenitors to promote their differentiation towards chondrogenesis.^28^ In sum, SMAD4 is vital for the commitment and the proper differentiation for the chondrocyte and osteocyte lineage formation.

SMAD4 plays the dual roles regarding ‘self‐renewal.’ The SMAD4 inhibition affects neither human embryonic stem cell self‐renewal nor the neuroectoderm formation,^14^ although this protein may play roles in the self‐renewal of hematopoietic stem cells.^18^, ^19^ SMAD4 may exert its different roles in self‐renewal of cells in a cell‐ and tissue‐specific manner.

SMAD4 also functions in ‘stem cell migration’ that is undertaken in two different levels: in intracellular and in the cell population. In the former, TGF‐β1 not only promotes the formation of gap junctions in chondrocytes via SMAD3/4 signalling pathways but also increases the cartilage precursor cell differentiation and chondrocyte proliferation, migration and metabolism.^29^ In cell population levels, the SMAD4/TGF‐β pathways promote cell migration, adhesion and cytoskeletal organization in different cells.^30^ In fact, these pathways involved in cell polarity are highly conserved whereby cells regulate the cytoskeletal organization above and beyond the subcellular organelle localization to facilitate cell proliferation and migration. SMAD4 regulates cell polarity in chondrocytes^31^ that is a process changing theshape, size, migration and orientation of the chondrocytes.

SMAD4 also plays in ‘stem cell maintenance’. The targeted ablation of SMAD4 in the epidermis increases the β‐catenin nuclear localization and c‐Myc activation that can deplete follicle stem cells. These suggest a critical role of SMAD4 in the normal maintenance of follicle stem cells.^32^ In osteoblasts, SMAD4 regulates hematopoietic stem cell fate and maintenance in a stage‐dependent manner.^33^

There is a snippet of information about how SMAD4 plays in the stem cell differentiation, maintenance and self‐renewal in bone biology; however, many aspects still need to be cleared in future studies by considering the chondrocyte and osteocyte as the target cells.

## 3 SMAD4 FUNCTIONS IN ANIMAL SKELETAL DEVELOPMENT

**FIGURE 2:**
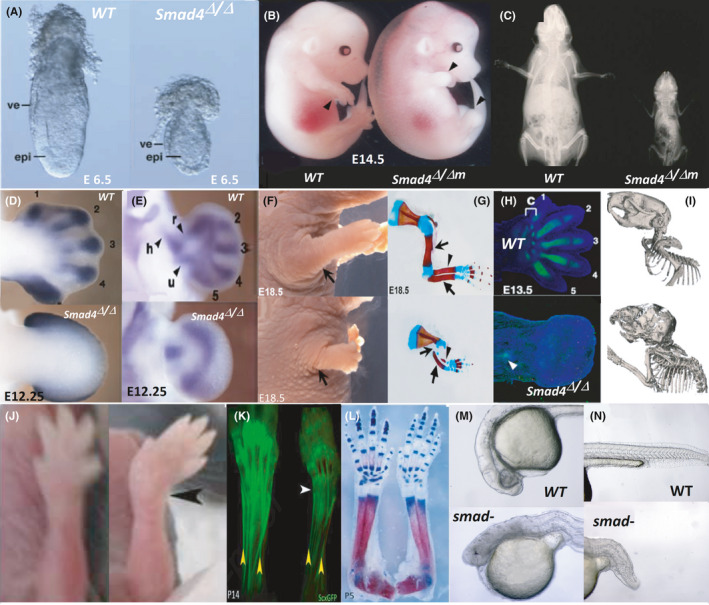
SMAD*4* mutations cause some important manifestations in animal models. (A) The entire deletion of SMAD*4* **(**SMAD*4* ^Δ/Δ^) causes failure in gastrulation in putative mice. The wild‐type (WT) and the mutated models of mouse embryos at E6.5 are shown. The mutation changes the epiblast (epi) epithelium and visceral endoderm (ve) section in affected mouse models. The figure is from Ref. [141]. (B) Bright‐field images of WT and SMAD*4^Δ^* ^/ΔM^ embryos at E14.5 reveal the short paddle‐like limb morphology in affected individuals. This morphology is extendable to both forelimb and hindlimb. The figure is from Ref. [28]. (C) The Skeletal phenotype of SMAD*4 ^F^* ^/^ *^F^*; *Osx1*‐*Cre mice* confirms the dwarfism and impaired extended osteogenesis. The figure is from Ref. [142]. (D) *Cyp26b1* marks the phalanx‐forming regions of all developing digit primordia in WT, while almost no differential expression is detectable in SMAD*4^Δ^* ^/^ *^Δ^* forelimb buds at E12.25. (E) The similar pattern was detected regarding the SOX*9* expression in limb region at E12.25. The figures are from Ref. [28]. (F) The impaired and short skeletal development of the SMAD*4* mutant mouse model. (G) Skeleton staining affirms such a hypothesis. In this figure, arrows, arrowheads and un‐notched arrows delineate humerus, radius and ulna respectively. The figure is from Ref. [44]. (H) The collagen type II distribution (green fluorescence) at E16.5 is shown in WT and mutant mouse models. In WT, collagen type II shows all developing limb skeletal elements, while in SMAD*4^Δ^* ^/^ *^Δ^* forelimb buds, a thin signal of collagen type II antibodies was detected (arrowheads). No boundary elements for fingers or other advanced structures were detected in the mutant model. (I) The three‐dimensional mouse computerized tomography scan showed the skeletal structures of 4‐week‐old mouse models and highlighted the underdeveloped and hypomineralized sections as well as severe hypomineralization of the craniofacial and axial skeleton in a mutant mouse model. The figure is from Ref. [45]. (J) SMAD*4* mutant mouse displays forelimb abduction (black arrowhead) in comparison with the WT littermates. The figure is from Ref. [45]. (K) GFP‐labelled tendons in SMAD*4^ScxCre^* mutant showed thinner tendons than control at P5. This figure is from Ref. [45]. (L) SMAD*4^ScxCre^* limb skeletons show some abnormality in chondrogenesis and osteogenesis in addition to the tendons. The figure is from Ref. [45]. (M, N) The SMAD*4* morphants manifest a shortened body due to the BMP inhibition, as the most severe form of manifestation. The SMAD4 mutants showed anterior truncation along with the crooked shortened body axis and absence of floorplate. The figure is from Ref. [46]. All figures were used with registered permission.

Knockdown of SMAD4 in mice causes early embryonic death,^34^, ^35^ ie SMAD4^/^mouse die before E7.5 mainly due to extensive gastrulation defects^34^ (Figure 2A). To assess postnatal complications, tissue‐specific SMAD4 knockout (KO) mice have been generated. The SMAD4 dysregulation is correlated with different embryonic developmental disorders,^36^ impairment in the skeletal muscle differentiation and regeneration,^37^ deficiency in stem cell pluripotency^38^ and impaired nervous system development.^39^ Herein, we only discuss those complications that are related to the skeletal development.

SMAD4 controls gene regulatory networks in early limb buds, most of which play roles in the anterior limb bud mesenchyme.^28^ SMAD4^/^mouse showed an impairment of cholesterol biosynthesis in the limb bud,^40^ explaining why sonic hedgehog (SHH) signalling is modulated in SMAD4‐*KO* mice.^40^ The SMAD4 conditional deletion (SMAD*4*‐*cKO*) impairs cartilage elements in limbs, verifying that SMAD4 is necessary for the cartilage formation. Besides, chondrocyte‐specific SMAD*4*‐*KO* mice manifest dwarfism and impaired growth plate organization^41^ (Figure 2B,C).

While SMAD4 inactivation may not change the early distribution of SOX9 in the limb bud, it disrupts the formation of SOX*9*‐positive digit ray primordia. These processes are associated with extensive chondrogenesis and osteogenesis. SMAD4 is required for the normal development of anterior and posterior digit ray primordia. SMAD4‐affected SOX9 expression affects the downstream gene expression and development, so it causes general loss of tissue organization as well as the redirection of mutant cells to nonspecific connective tissue^42^ (Figure 2D,E). In essence, SMAD4 is necessary to stimulating the formation of the SOX*9*‐positive digit ray primordia and promoting the aggregation and chondrogenic differentiation of all limb skeletal elements. However, SMAD4 may be necessary for the early steps of cartilage formation independent of SOX9 expression,^43^ verifying the tissue‐specific SMAD4 regulation.

SMAD4 is abundantly expressed in prehypertrophic and hypertrophic chondrocytes. Any gene perturbation in chondrocytes may result in a dwarfism phenotype with a haphazardly arranged growth plate featured by the expanded resting zone of chondrocytes, lowered chondrocyte proliferation, quickening hypertrophic differentiation, increased apoptosis and ectopic bone collars in the perichondrium (Figure 2F,G). Moreover, decreased expression of Indian hedgehog/parathyroid hormone‐related protein (Ihh/PTHrP) signalling‐related molecules was observed in SMAD4 mutant mice.^41^ Deficient mice also manifest stunted limbs (ie hindlimb or forelimb) along with a substantially reduced expression of the chondrocyte differentiation markers such as *Col2a1*, *Col2a2* and *Acan* in humerus at mid‐to‐late gestation^44^ (Figure 2H). The most important manifestation was the absence of stylopod elements and failure of chondrocyte hypertrophy in the humerus^44^ (Figure 2G). The expression of *Col10a1*, *Panx3* and RUNX2 was decreased in SMAD4^−/−^ mice.^44^

In animal models, SMAD*4*‐inactivating mutations cause dwarfism and spontaneous fractures (Figure 2I). SMAD4 controls the maturation of skeletal collagen and osteoblast survival. This protein is also necessary for matrix‐forming responses. Although affected bones in SMAD*4^Δ^* ^/^ *^Δ^* mice show fully differentiated osteoblast markers, they did not have multiple collagen‐processing enzymes, particularly lysyl oxidase that is regulated by SMAD4 and RUNX2.

In addition to impaired chondrocytes and osteocytes, the SMAD4 depletion impresses the scleroaxis and coordinated tendon elongation, eg SMAD*4ScxCre* mice develop a joint contracture that is stochastic in the direction and is exacerbated with age^45^ (Figure 2J–L). This can substantiate the vital roles of SMAD4 in the initiation and fixation of the chondrocyte and bone development.

The SMAD*4* morphant zebrafish manifests severely impaired growth and notochord defects in comparison with SMAD*2*/*3a*/*3b* morphants. These severe phenotypes were imputed to the pleiotropic permissive functions of SMAD4.^46^ This study also showed that SMAD*4* morphant caused a more severe phenotype in the spinal cord (compared with other SMAD genes), verifying its important roles in neurogenesis, chondrogenesis and osteogenesis (Figure 2M,N). Besides, Sun et al. showed that SMAD4 along with other proteins initiate a positive feedback loop of BMP signalling within the embryo that led to the normal dorsoventral patterning.^47^

## 4 SMAD4 REGULATES APOPTOSIS

The importance of the cell‐programmed death or apoptosis in skeletal tissues and its functional relationship with the chondrocyte and osteocyte development has been thoroughly investigated.^48^, ^49^ Osteogenic lineage cells (eg osteoblasts and osteocytes) in addition to the osteoclastic cells are influenced by apoptosis.^49^, ^50^ There is a controversy regarding the roles of SMAD4 in apoptosis; although some studies attribute the apoptotic roles to this protein, recent investigations ascribe an apoptotic inhibitory function to this protein. For instance, SMAD4 contributes to follicular atresia by suppressing granulosa cell apoptosis.^51^

TGF‐β induces the SMAD4‐dependent epithelial‐to‐mesenchymal transition followed by apoptosis in colorectal cancer cells.^52^ Regarding the skeletal development, the protective roles of SMAD4 against apoptosis have been identified,^53^ eg the depletion of SMAD4 in chondrocytes resulted in a higher rate of apoptosis and ectopic bone collars in perichondrium that disorganizes growth plate cartilage.^41^ In other words, the SMAD4‐mediated TGF‐β signalling pathway suppresses the chondrocyte hypertrophic differentiation and maintains the normal organization of chondrocytes in growth plate.^41^ Likewise, osteoblasts overexpressing *SMAD4* show increased apoptosis. SMAD4 plays an important function by regulating osteoblast/osteocyte viability and bone homeostasis.^54^ Apoptosis is essential for the differentiation and bone homeostasis,^55^ ie osteoblast apoptosis promotes the osteoclastogenesis and bone resorption, which is a vital process for bone homeostasis. Enhancing osteoblast or osteocyte viability sets the stage for protection against osteoporosis, ie SMAD4 is essential for recovery from pathological bone conditions.^54^, ^56^ Osteoclast‐specific SMAD*4*‐*cKO* mice exhibited reduced bone mass with increased osteoclast formation.^57^

Yang et al. showed that chondrocyte‐specific SMAD*4*‐*cKO* increased apoptosis.^58^ The SMAD4 inhibition in osteoarthritis has also been documented,^59^ revealing the protective roles of SMAD4 against apoptosis and its pivotal roles in inducing the chondrocyte differentiation and proliferation.

## 5 SMAD4 FUNCTIONS THROUGH DIFFERENT SIGNALLING PATHWAYS

### 5.1 TGF‐β signalling axis

TGF‐β needs the SMAD family to function normally, and it stimulates a variety of physiological processes such as tissue repair, cell growth, cell differentiation and cell proliferation.^60^, ^61^ TGF‐β activates two types of receptors including the activin receptor‐like kinase 1 (ALK1) and ALK5. TGF‐β stimulates (using ALK1) or inhibits (via ALK5) the migration and proliferation of endothelial cells.^62^

**FIGURE 3:**
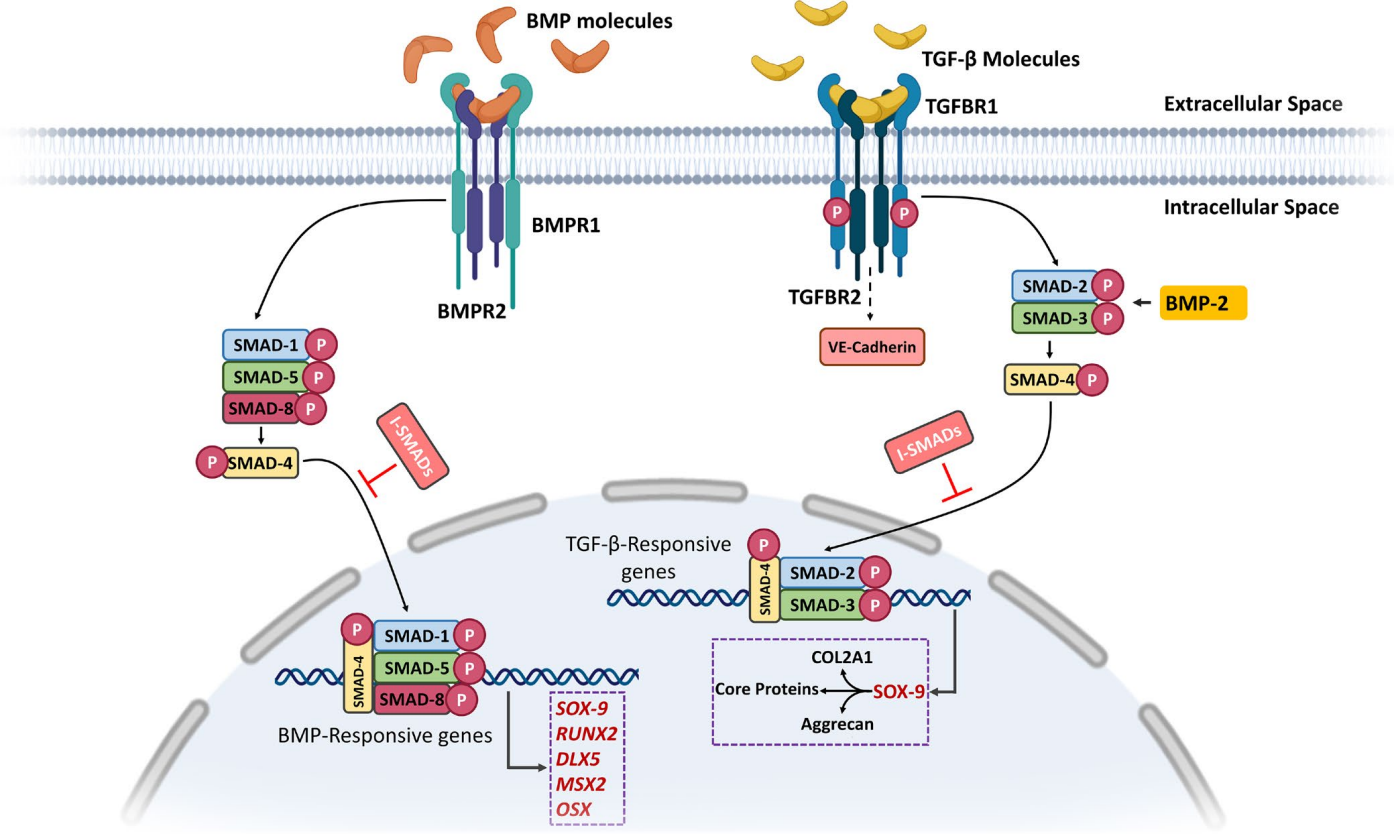
TGF‐β/SMAD and BMP signalling in skeletal development. TGF‐βs and also BMPs bind to their specific cell membrane receptors (receptor types II) and recruit receptor types I. Upon binding, TGF‐βRs phosphorylate the downstream R‐SMADs, while BMPRs phosphorylate the SMAD1/5/8, as a different group of R‐ SMADs. By binding to co‐SMADs—SMAD4, R‐SMADs form a complex and as a transcription factor activates the downstream TGF‐β and BMP‐responsive gene expression that are playing important roles in the skeletal development. I‐SMADs, ie SMAD6/7, negatively regulate TGF‐β/SMAD signalling. The figure is redrawn from Ref. [143]

Seven TGF‐β superfamily type I receptors (are also known as ALKs) have been identified so far. ALK1, 2, 3 and 6 contribute to signalling by activating the SMAD1, 5 or 8.^62^ On the other hand, ALK4, 5 and 7 transduce signals by phosphorylating the SMAD2/3.^62^ TGF‐β binds to constitutive active type I and II receptors that are followed by the phosphorylation of R‐SMADs. A complex of two R‐SMADs and SMAD4 is formed that shuttles to the nucleus to regulate the expression of target genes (Figure 3). In chondrocytes and other endothelial cells, TGF‐β handles signalling using ALK1, in which SMAD1/4/5/8 axis is activated. TGF‐β signalling via ALK1 leads to different gene activation and cellular responses than ALK5 signalling.

Transcriptional intermediary factor 1γ (*TIF1γ*; also called *Ectodermin*/*PTC*/*RFG7*/*TRIM33*) is a transcriptional cofactor competing with *SMAD2*/*3* to bind with SMAD4^63^; however, its functions in chondrogenesis and osteogenesis yet are still blanketed in mystery. TIF1γ regulates the TGF‐β signalling pathway by controlling SMAD4.^63^ TIF1γ influences the stability of SMAD4 by promoting its degradation through the ubiquitin proteasome in humans. TIF1γ serves as a RING‐finger ubiquitin ligase for SMAD4, and the implicated monoubiquitination performed by TIF1γ stimulates its nuclear export and suppresses the formation of SMAD nuclear complexes.^64^ Therefore, TIF1γ deficiency causes the SMAD4 nuclear localization. An inverse relationship exists between the levels of TIF1γ and SMAD4 in the pancreatic cells.^65^ TIF1γ mediates the differentiation responses, while SMAD4 modulates the antiproliferative responses in connection with SMAD2/3^65^; however, its roles in the skeletal development still need further clarification.

SMAD3/4 complex promotes the transcription of SNAI1 protein and reduces the expression of the epithelial junction protein E‐cadherin in the SMAD4‐dependent pathway. E‐cadherin is essential for the normal development of the skeletal structures.^66^

**FIGURE 4:**
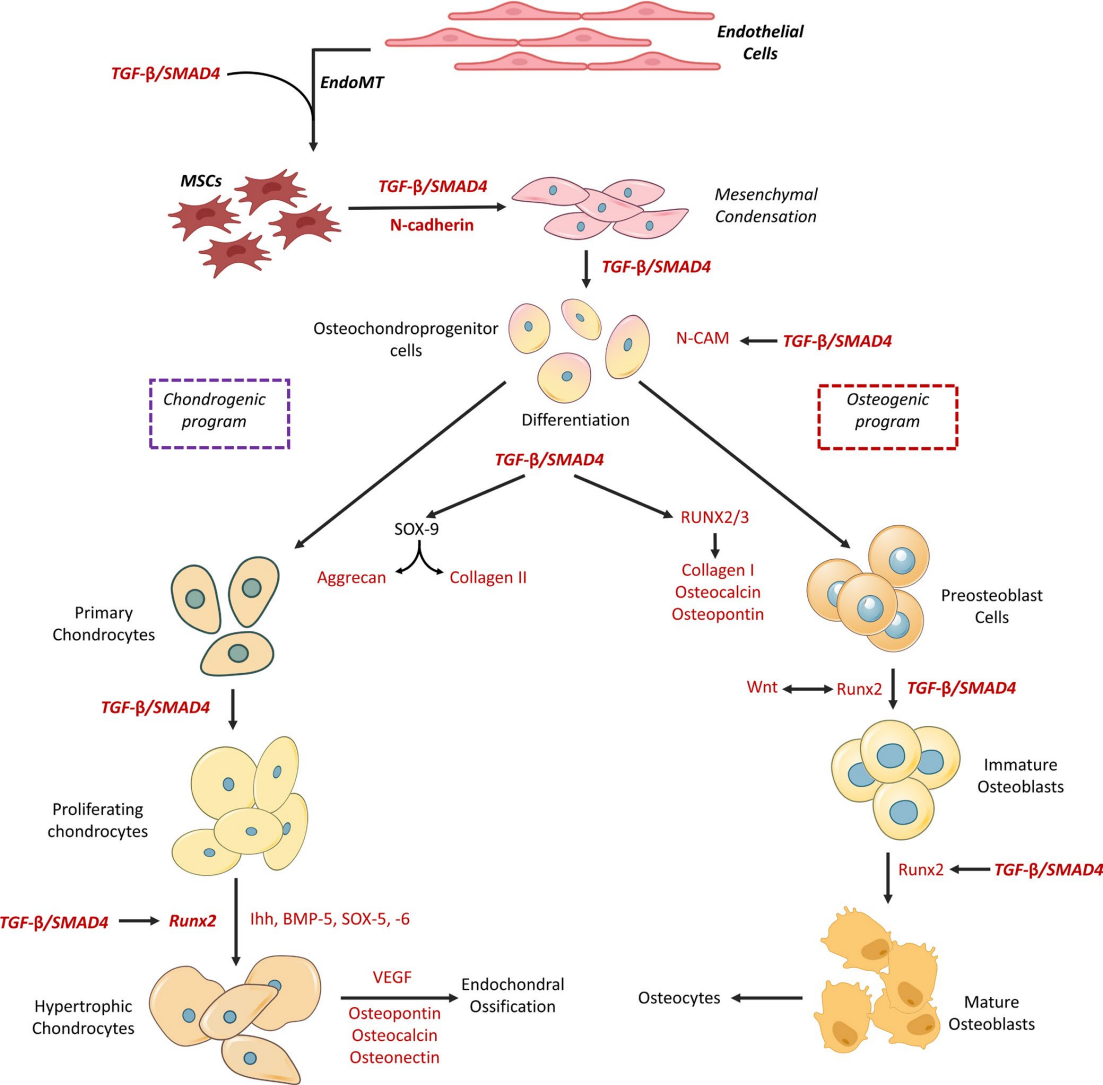
TGF‐β/SMAD4 contributes to chondrogenesis and osteogenesis. After condensation, mesenchymal stem cells (MSCs) make the osteochondroprogenitor cells that in turn can be committed to being differentiated into osteogenic or chondrogenic cell lineages. TGF‐β/SMAD4 plays roles from MSCs to osteocytes and endochondral ossification. EndMT: endothelial‐to‐mesenchymal transition; BMP, bone morphogenetic protein; N‐CAM, neural cell adhesion molecule; Ihh, Indian hedgehog; VEGF, vascular endothelial growth factor; RUNX2/3: runt‐related transcription factor 2/3; TGF‐β, transforming growth factor‐β; SOX: SRY (sex‐determining region Y)‐box. The figure is redrawn from Ref. [144, 145]

Similarly, TGF‐β exerts its functions through SMAD4, especially by stimulating the downstream gene expression, eg *RUNX2*, *DLX5*, *MSX2*, *OSX* and *SOX9*.^67^ As depicted in Figure 4, the downstream genes can, in turn, contribute to the skeletal development. In essence, TGF‐β plays multiple roles in chondrogenesis, mesenchymal condensation, chondrocyte proliferation, extracellular matrix deposition and terminal differentiation.^68^

Using SMAD4, TGF‐β sets stages for the normal development of chondrogenesis from MSCs; in other words, TGF‐β stimulates the expression of N‐cadherin, so enhancing cell adhesion and causing cell condensation during chondrogenesis. N‐cadherin promotes the expression of *ACAN* and *COL2A1*, a process that can be attributed to the direct stimulation by TGF‐β or the upregulation of *SOX9* as a downstream gene (Figure 4). SOX9 plays as an enhancer for the *COL2A1* promoter.^1^ SMAD4 is critical for the TGF‐β‐driven upregulation of N‐cadherin^69^ (Figure 4), and so, TGF‐β promotes the initial stages of chondrogenesis.

### 5.2 Bone morphogenic protein

As important growth factors, BMPs contribute to the chondrocyte‐to‐osteocyte and craniofacial development. BMP ligands bind to type II BMP receptors (BMPRII), subsequently followed by recruiting type I BMP receptors (BMPRI) to form a heterogeneous tetramer. BMPRII phosphorylates itself in addition to the BMPRI in the tetramer. These events give rise to transduce signals into the cytoplasm by activating the SMAD‐dependent pathway (as canonical BMP signalling) or the SMAD‐independent pathway (as noncanonical BMP signalling).^70^ In the former, BMPRI phosphorylates SMAD1, 5 or 8, which forms transactivator with SMAD4 to activate the transcription of downstream target genes (Figure 3). In the SMAD‐independent pathway, the signal is transferred through the phosphorylation of p38, Erk or JNK.

Three major classes of BMPRIs have been identified—including ALK2, ALK3 and ALK6—that directly stimulate the intracellular BMP signalling (Figure 3). BMP signalling is controlled in both intracellular and extracellular manners. While in the former, SAMD4 interacts with SMAD6 or 7 to exert its inhibitory roles, in an extracellular manner, secreted antagonists (eg Noggin) play the central roles. These antagonists contribute to chondrogenesis, osteogenesis and joint formation during the embryonic development. BMP‐SMAD4 signalling is essential for cell proliferation in the anterior palatal mesenchyme^71^ and also for the osteogenesis in the posterior palatal mesenchyme.^71^, ^72^ Any perturbation of *Bmpr1a* in palatal mesenchyme may result in cleft palate manifestation. In fact, the enhanced BMP‐SMAD4 signalling gave rise to premature osteogenic differentiation in palatal mesenchyme^71^; however, in the *Osr2*‐*creKI*‐SMAD*4^f^* ^/^ *^f^* mice, complete cleft palates with the compromised cell proliferation and osteogenesis were observed.^73^ This can underscore that the regulation of the BMP‐SMAD4 axis was more complicated than previously thought.

BMP‐SMAD4 signalling also regulates mesenchymal condensation during the skeletal development, probably utilizing a SOX9‐independent mechanism^43^; therefore, further investigations should be conducted to show how downstream modulators contribute to BMP–SMAD4 signalling in precartilaginous mesenchymal condensation. It has been also elaborated that BMP signalling promotes the osteoblast differentiation through both SMAD4‐ and mTORC1‐dependent mechanisms,^74^ suggesting that the interactions between BMP‐SMAD4 and other modulators are somehow complicated. BMP2/SMAD4 promotes the expression of several downstream mineralization‐related genes but does not contribute to the subsequent activation of protein anabolism.^74^

Moreover, two phases of high BMP activity are required for the normal limb bud development. Using a mouse model, BMP has been verified to activate *Grem1* expression via SMAD4. *Grem1* is a critical node in the SHH/GREM1/FGF feedback signalling axis.^75^

In a nutshell, BMPs through SMAD4 can (i) control the chondrogenesis, osteogenesis and joint formation during the embryonic development, (ii) regulate cell proliferation and osteogenesis in palatal mesenchyme, (iii) regulate premature osteogenic and osteoblast differentiation, eg in palatal mesenchyme, (iv) regulate precartilaginous mesenchymal condensation and (v) modulate the expression of different downstream mineralization‐related genes.

### 5.3 Wnt signalling pathways

TGF‐β and Wnt signalling pathways are interrelated in most cells and tissues. As one of the important regulators in different developmental processes, especially in the skeletal development, the Wnt family contains at least 22 cysteine‐rich, secreted glycoproteins. Of these, Wnt‐4, −5a, −5b and −14, along with the secreted frizzled related protein (a Wnt antagonist), are often expressed in the developing skeleton. While Wnt‐4 inhibits the initiation of chondrogenesis and promotes the terminal chondrocyte differentiation, Wnt‐5a/5b stimulates early chondrogenesis and suppresses the terminal differentiation. Wnt‐14 serves at the initial steps of the joint development.

**FIGURE 5:**
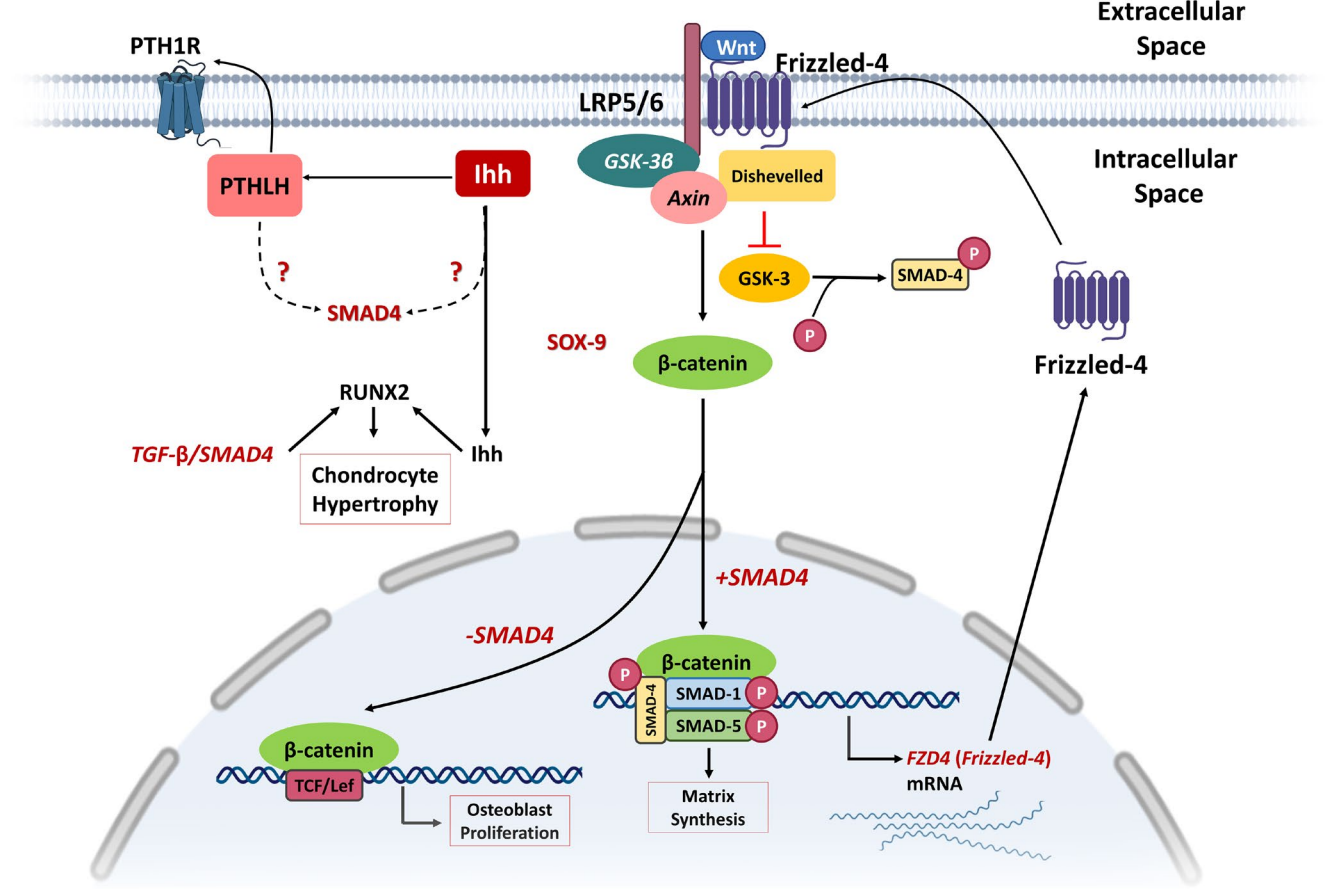
The schematic representation of the Wnt/β‐catenin pathway in skeletal development. Upon ligation Wnts to their specific receptors that contain the frizzled proteins (FZD4) and LRP5/6, the cytoplasmic protein dishevelled is promoted to induce the inhibition of GSK3β. Then, as a transcription factor, β‐catenin binds to the TCF/LEF transcription factors and leads to the transcription of downstream target genes. Competitive recruitment of β‐catenin by SMAD4 controls the balance between proliferation and matrix synthesis of osteoblasts. SMAD4 lacking can put the availability of β‐catenin on increase to transactivate Tcf/Lef, hence enhancing ‘proliferation’ in response to canonical Wnt signalling. The figure is redrawn from Ref. [77]

Wnt signalling pathways are mainly categorized into two different pathways including the β‐catenin‐dependent pathway or canonical Wnt/β‐catenin signalling, and the β‐catenin‐independent pathway or noncanonical one. The canonical Wnt/β‐catenin signalling is promoted by binding Wnts to the Frzb/co‐receptor (lipoprotein receptor‐related protein‐LRP‐5/6) complex, resulting in some conformational changes in the downstream molecule complex that mainly consists of dishevelled, Axin, glycogen synthase kinase 3β (GSK3β) and β‐catenin (Figure 5). GSK3 phosphorylates the linker regions of SMAD4 and inactivates TGF‐β signalling.^76^ Besides, SMAD4 promotes the expression of *frizzled*‐*4 (FZD4)* and activates FZD4‐dependent Wnt signalling in granulosa cells^51^; however, this pathway is still unclear in the skeletal development. Interestingly, TGF‐β/SMAD4 and Wnt signalling pathways modulate apoptosis. Du et al. suggested that the apoptotic roles of SMAD4 are due to the maintained expression of *FZD4* at both the transcriptional and post‐transcriptional level mainly through a complex regulatory network.^51^ In essence, by phosphorylation of SMAD4, GSK3β not only controls TGF‐β signalling but also regulates the expression of *FZD4*. Additionally, the competitive recruitment of β‐catenin with SMAD4 modulates the balance between proliferation and matrix synthesis in osteoblasts.^77^

The noncanonical Wnt pathway is also important in the skeletal development, although there is some controversy about the roles of this pathway in vertebrates. Different intercellular signalling molecules play roles such as inositol triphosphate‐intercellular calcium, mitogen‐activated protein kinases (MAPKs) and G‐protein RhoA/Rho‐associated kinase. MAPK activated by TGF‐β functions as an effector mediating SMAD4‐independent TGF‐β signalling during the tooth and palate development.^78^ TGF‐β induces the expression of a downstream noncoding RNA (miR‐155) and promotes the promoter activity through SMAD4. Aberrant expression of miR‐155 substantially decreases RhoA protein and disorganizes the tight junction formation^79^ in breast cancer cells. An interaction that exists between TGF‐β/SMAD4 and RhoA may be extended to bone biology.

SMAD4 can target SFRP2, which is a Wnt inhibitor. SFRP2 is downregulated in the SMAD*4^Δ^* ^/^ *^Δc^* forelimb buds like other Wnt antagonists such as DKK1 or WIF1.^80^ The inhibition of Wnt signalling in limb buds of SMAD*4* mutant shows a direct and/or indirect regulation system accomplished by SMAD4.

### 5.4 Runt‐related transcription factor 2

RUNX2 stimulates the early commitment of MSCs to the osteochondrogenic progenitors^81^ and controls the expression of osteoblast markers such as osteocalcin, osteopontin, bone sialoprotein, collagenase 3, osteoprotegerin and α1(I) and α1(II) collagens.^82^, ^83^ Thanks to the stimulation of the Indian hedgehog (Ihh), RUNX2^+^ osteoprogenitors are developed into chondrocytes^84^ (Figure 4). RUNX2 directly targets *Ihh* that is expressed in prehypertrophic chondrocytes and is necessary for chondrocyte hypertrophy. Furthermore, the RUNX transcription factors, eg RUNX2 and RUNX3, control and orchestrate the chondrocyte hypertrophic differentiation and proliferation.^85^

The RUNX2 is regulated in the transcription, translational and post‐translational levels. Yan et al. showed that SMAD4 regulates the chondrocyte hypertrophy by upregulating RUNX2 during the skeletal development.^44^ SMAD4 also interacts with RUNX2 to induce the expression of other osteoblast genes. For example, RUNX2 directly regulates COL10A1 that is a hypertrophic chondrocyte‐specific marker. On the other hand, SMAD4 promotes RUNX2 degradation in a ubiquitin proteasome‐dependent manner, a process that may affect the osteoblast differentiation. In essence, SMAD4 controls the functions and the expression of RUNX2 and also other osteoblast genes in a feedback loop. Upon external stimulation, the cytoskeleton regulates the activation of SMAD4/RUNX2 signalling in MSCs, so can in turn impress the stem cell commitment.^86^ Therefore, inhibiting RUNX2 and SMAD4, eg using RNA interference, is a powerful approach to prevent or treat heterotopic ossification.^87^

### 5.5 Notch signalling

Notch signalling is vital for the normal embryonic development, coordinated tissue homeostasis and stem cell maintenance.^88^ Notch functions on chondrogenesis using both CSL (CBF1, suppressor of hairless, Lag‐1)‐dependent and CSL‐independent mechanisms.^89^, ^90^ Upon activation, Notch signalling controls a balance between the chondrogenic proliferation and differentiation at the early stages of somite compartmentalization and the long bone development.^89^ Notch promotes the final differentiation of the osteoblast progenitors but seems to have no obvious effects on mature osteoblasts.^91^ It also inhibits the chondrogenic differentiation by suppressing the activity of *COL2A1* promoter and the expression of *SOX9*.^92^ Notch regulates *cartilage link protein 1* (*Crtl1*) as a target of SOX9.^93^ In osteoblasts, Notch signalling plays a dual role to either suppress or induce the osteoblastic differentiation. Notch signalling has been demonstrated *in vivo* to inhibit the osteoblastic differentiation by suppressing both early and late differentiation markers as in collagen type 1, RUNX2, alkaline phosphatase and osteocalcin.^94^, ^95^

SMAD4 helps to keep the cerebrovascular endothelial cell‐pericyte interactions stable by modulating the transcription of N‐cadherin by affecting its promoter. This mechanism links TGF‐β/SMAD4 and Notch signalling and maintains cerebral vascular integrity.^96^ The thrust of these results is that SMADs form a complex with Notch intracellular domain and CSL. TGF‐β/BMPs improve the recruitment of the complex to the CSL‐binding site to transactivate the *N*‐*cadherin* promoter.^96^ A reciprocal interaction exists between SMAD4 and Notch signalling pathway, eg downregulation of Notch1 and c‐MYC in SMAD*4*‐deficient hematopoietic mice can enrich hematopoietic stem cells^18^; however, these functions are still needed to be clearly shown in bone biology.

## 6 SMAD4 CAN BE CONTROLLED EPIGENETICALLY

### 6.1 Noncoding RNAs

Noncoding RNAs take part in the skeletal development.^1^ Although a variety of noncoding RNAs have been identified so far, the discovery of microRNAs (called miRNAs or miRs) and later long noncoding RNAs (lncRNAs) revolutionized our knowledge of these critical functions of these molecules in the skeletal development.^97^, ^98^ MiRNAs (~22 nucleotides) encourage the destruction or inhibit the translation of target mRNAs, so playing a vital role in the developmental and physiological processes.^1^, ^98^, ^99^, ^100^, ^101^

Overexpression of miR‐483 downregulates SMAD4.^102^ MiRNA profiling of developing human cartilage of limb revealed that this miRNA is expressed at high levels in chondrocytes that were in the proliferation and differentiation stages. MiR‐483 is downregulated in hypertrophic chondrocytes, indicating its possible roles in regulating the hypertrophic differentiation.

MiR‐29b regulates homeostasis of chondrogenesis and enhances the hypertrophic phenotype. In fact, this miRNA is a fundamental regulator of the chondrocyte phenotype from murine marrow‐derived MSCs, and it is a target for articular cartilage repair.^103^ While the expression of miR‐29b was at a low level during the early chondrogenic development, it increased during chondrogenic hypertrophy. SMAD4 contributes to the TGF‐β‐regulated transcriptional upregulation of miR‐29 in senescent mouse embryonic fibroblasts; however, this signalling pathway remains to be discovered in the skeletal development.^104^

MiR‐224 directly regulates SMAD4 and results in the inhibition of osteoblast differentiation.^105^ Besides, TGF‐β1/SMAD4 signalling affects the osteoclast differentiation by regulating miR‐155 expression, and using miR‐155 as a therapeutic target for osteoclast‐related disorders has shown a lot of promise.^106^ MiR‐144‐3p also inhibits the osteogenic differentiation of bone MSCs by suppressing SMAD*4* expression.^107^

Furthermore, miR‐146a may make a contribution to osteoarthritis by increasing the VEGF levels and by suppressing SMAD4 in cartilage cells.^53^ This miRNA participates in human chondrocyte apoptosis caused by mechanical injury.^108^ Osteoporosis is a prevalent disease that threatens patients’ quality of life, particularly elderly patients. The upregulation of miR‐146a decreases the *SMAD4* expression in osteoporosis patients.^109^ Similarly, by targeting SMAD4, miR‐449c‐5p has been suggested as a novel inhibitory regulator of the osteogenic differentiation in valve interstitial cells. This miRNA also is a new therapeutic agent for calcific aortic valve disease.^110^ Xie et al. demonstrated that antagomiR‐1323 therapy may boost fracture healing in a rat model of femoral fracture. This miR‐1323 negatively controls *BMP4* and *SMAD4* expression in human atrophic nonunion fractures, supporting that miR‐1323/BMP4 and miR‐1323/SMAD4 are a novel therapeutic target for atrophic nonunion fractures.^111^ Although the different roles of miRNAs and their functions on SMAD4 have been documented, profiling miRNA is highly recommended during the chondrocytes‐to‐osteocyte development.

### 6.2 Long noncoding RNAs

lncRNAs (with a total length of >200 nt) play different roles during the development of different organs. In general, plenty of lncRNAs have been identified to take part in chondrogenesis and osteogenesis.^1^, ^112^ For example, lncRNA UCA1 stimulates the chondrogenic differentiation from MSCs through miRNA‐145‐5p/SMAD5 and miRNA‐124‐3p/SMAD4.^113^

lncRNA DANCR promotes the chondrogenic differentiation in synovial MSCs by stimulating the miR‐1305/SMAD4 axis.^114^ As the expression of DANCR increases, it results in the decreased expression of miR‐1305 that in turn negatively regulates the expression of SMAD4.

lncRNA MALAT1 regulates the osteogenic differentiation via three key mechanisms: firstly, MALAT1 modulates Osterix overexpression in human MSCs and subsequently binds to and inhibits miR‐143 in order to induce the osteogenic differentiation. Secondly, MALAT1 functions as a sponge for miR‐204 and boosts the expression of SMAD4. These trigger the bone formation and subsequent mineralization. MiR‐204 functions as a positive regulator of the osteogenic differentiation of human aortic valve interstitial cells.^115^ Thirdly, using osteosarcoma cell models, MALAT1 has been identified to promote metastasis and proliferation by inducing miR‐144‐3p, which in turn targets SMAD4.^116^

lncRNA AWPPH stimulates cell proliferation, autophagy and migration, and inhibits apoptosis in bladder cancer by suppressing SMAD4.^117^ This lncRNA upregulates RUNX2 and in turn contributes to the development of nontraumatic osteonecrosis of the femoral head.^118^ While AWPPH is downregulated in osteoporosis, in normal tissues, it regulates the balance between type I collagen α1 and α2 ratio,^119^ which is vital for the osteocyte maturation and bone homeostasis.

Besides, lncRNA MFAT1 controls *Tgfbr2*/ SMAD*4* expression by sponging miR‐135a‐5p that gives rise to the TGF‐β pathway activation in skeletal muscle fibrosis. This provides a novel‐promising therapeutic option against skeletal muscle fibrosis.^120^ Silencing MFAT1 attenuated the expression of TGFBR2 and SMAD4 through miR‐135a.

### 6.3 DNA methylation

DNA methylation is one of the important molecular mechanisms whereby the skeletal development is controlled. For instance, Aitchison et al. attributed the low expression of *SMAD4* to its promoter's methylation status in advanced prostate cancer. This suggested that SMAD4 is regulated by DNA methylation.^121^ Interestingly, the downregulation of *SMAD4* caused by promoter methylation can, in turn, bring about the impairment of TGF‐β signalling. This process is often observed in the growth suppression of Barrett's oesophageal epithelium metaplasia.^122^ There is actually little to no information about the methylation status of the *SMAD4* during embryogenesis, chondrogenesis and osteogenesis.

### 6.4 Histone modifications

During the skeletal development, histone modifications regulate or even boost gene expression. For example, overexpression of histone deacetylase 4 (HDAC4) inhibits the expression of several downstream genes related to the TGF‐β signalling pathway such as *BMP6*, *SMAD4*, *SMAD6*, *inhibitor of DNA binding 1* and *TGF*‐*β2*. HDAC4 controls *SMAD4* expression by inducing the deacetylation of histone H3 in promoter regions.^123^ SMAD3/SMAD4 complex cooperates with the HDACs to modulate the androgen receptor acetylation,^124^ a process that is vital for the bone development and homeostasis (reviewed in Ref. [125, 126]).

Long et al showed that ‘Histone SUMOylation’ represses SMAD4 transcriptional activity^127^ and regulates its stability. SUMOylation of SMAD4 is necessary for allowing this protein to bind other transcriptional factors such as DAXX,^128^ which inhibits SMAD4‐mediated transcriptional activity by direct interaction with the sumoylated SMAD4, so indirectly regulates TGF‐β signalling.

The conversion into MSCs via the endothelial‐to‐mesenchymal transition can be triggered in chondrocytes. SIRT1 inhibits the TGF‐β‐induced endothelial‐to‐mesenchymal transition (that can protect against fibrosis) by the deacetylation of SMAD4.^129^ The upregulation of SIRT7 increases the expression of E‐cadherin, while decreases the expression of mesenchymal markers. Besides, overexpression of SIRT7 also decreases the level of acetylated SMAD4 in oral squamous cell carcinoma cells.^130^ These studies introduce a good starting point for future investigations.

## 7 CONCLUSIONS AND FEATURE PERSPECTIVES

The skeletal development is a complex procedure in which different signalling pathways play roles. More recently, some important steps have been taken forward to investigate this process, although there is a long journey to pave. Although some investigations have been conducted to show how SMAD4 functions during the bone development, some aspects are still needed to be unveiled.

In this review, we discussed why SMAD4 is critical for the skeletal development and bone homeostasis; however, there is a snippet of information about the functions of this protein during the skeletal development. Herein, we summarized some fundamental findings to show the molecular mechanisms whereby SMAD4 contributes to chondrogenesis and osteogenesis. Assuredly, in the future, more investigations can remove the veil of ignorance and answer to some open‐ended questions; for example, exosomes, membrane‐bound extracellular vesicles that carry the specific molecules,^131^ contribute to the skeletal development^1^ and bone repair^132^, ^133^, ^134^; however, little is known about how exosomal SMAD4 or even related miRNAs and lncRNAs play roles during the skeletal development. Besides, it has been identified that taking medicines can also affect the expression and function of SMAD4, eg some studies discovered that valproic acid inhibited the *SMAD4* expression by monoubiquitination in prostate carcinoma.^135^ By inducing apoptosis, valproic acid decreases the number of chondrocytes.^136^ In fact, the monoubiquitination of SMAD4 may interfere with normal embryogenesis, chondrogenesis and osteogenesis; however, it is still unclear how other medicines can affect this process. Likewise, it is not crystal clear how other noncoding RNAs, eg piwiRNA, circRNA, as well as environmental modulators, affect the skeletal development and how SMAD4 functions during these events. In fact, the expression profile of miRNAs and lncRNAs during different stages of the skeletal development can somehow show how SMAD4 contributes to the development. Also, little is known about the potential therapeutic applications of SMAD4 in human diseases and bone fractures. As discussed, some alternations such as phosphorylation, methylation, and ubiquitination may play important roles in regulating SMAD4 activities and functions; however, we do not know exactly how such alternations take part in the chondrocyte and bone development, bone repair and human bone‐related disorders.

Moreover, SMAD4 has been recently employed as a therapeutic target in a variety of diseases such as pancreatic adenocarcinoma cells,^137^ colorectal cancer^138^ and fibrosis^139^; however, we do not know whether this protein can be used as a therapeutic agent/target for cartilage‐ and bone‐related disorders or not. We believe that the future investigations can answer these kinds of questions. Additionally, there are plenty of reports demonstrating that SMAD4 bestows some features to the putative stem cells, proposing that SMAD4 and other members of this family may potentially be used to reprogram the stem cells. This can in turn provide valuable information about the biological aspects of bone biology, which will pave the way to utilize this for therapeutic purposes.

## CONFLICT OF INTEREST

The authors declare no conflict of interest.

## AUTHOR CONTRIBUTIONS

**Katayoon Pakravan:** Writing – review & editing. **Ehsan Razmara:** Conceptualization; Visualization; Writing – original draft; Writing – review & editing. **Bashdar Mahmud Hussen:** Writing – review & editing. **Fatemeh Sattarikia:** Writing – review & editing. **Majid Sadeghizadeh:** Writing – review & editing. **Sadegh Babashah:** Conceptualization; Supervision; Writing – review & editing.

## Data Availability

The paper is exempt from data sharing.
